# The Platelet-to-Lymphocyte Ratio (PLR) as a Non-Invasive Biomarker for Cervical Malignancy in Conization Patients

**DOI:** 10.3390/life15060971

**Published:** 2025-06-18

**Authors:** Noémi Kalas, Verita Szabó, Balázs Vida, Zsófia Tóth, Lotti Lőczi, Barbara Sebők, Petra Merkely, Balázs Lintner, Nándor Ács, Attila Keszthelyi, Szabolcs Várbíró, Richárd Tóth, Márton Keszthelyi

**Affiliations:** 1 Department of Obstetrics and Gynecology, Semmelweis University, 1082 Budapest, Hungary; kalasnoemi@gmail.com (N.K.); szabo.verita@gmail.com (V.S.); vida.balazs.lajos@semmelweis.hu (B.V.); toth.zsofia99@stud.semmelweis.hu (Z.T.); keszthelyi.lotti.lucia@semmelweis.hu (L.L.); merkely.petra@gmail.com (P.M.); lintner.balazs.zoltan@semmelweis.hu (B.L.); acs.nandor@semmelweis.hu (N.Á.); toth.richard@semmelweis.hu (R.T.); 2 Workgroup of Research Management, Doctoral School, Semmelweis University, 1085 Budapest, Hungary; sebok.barbara23@gmail.com (B.S.); varbiroszabolcs@gmail.com (S.V.); 3 Department of Urology, Semmelweis University, 1082 Budapest, Hungary; keszthelyi.attila@semmelweis.hu; 4 Department of Obstetrics and Gynecology, University of Szeged, 6725 Szeged, Hungary

**Keywords:** platelet-to-lymphocyte ratio (PLR), cervical cancer, systemic inflammation, HPV DNA, biomarkers, ROC analysis, invasive carcinoma, risk stratification

## Abstract

Background: Cervical cancer continues to pose a significant global health challenge, particularly in low-resource regions with limited access to advanced diagnostics. Cervical conization can occasionally uncover invasive carcinoma in patients initially suspected of having only pre-invasive lesions. This study assessed the platelet-to-lymphocyte ratio (PLR) as a potential predictive biomarker for identifying invasive disease in patients undergoing a loop electrosurgical excision procedure (LEEP). Methods: A retrospective study was conducted on 371 patients who underwent LEEP conization for cervical dysplasia. Preoperative PLR values were collected and compared across final histopathological categories (negative, low-grade, high-grade, invasive carcinoma) using the Kruskal–Wallis test, followed by Mann–Whitney U tests for pairwise comparisons. Receiver operating characteristic (ROC) analysis was used to evaluate diagnostic accuracy. Results: PLR values above 7.7 were significantly associated with HPV positivity, increasing with histopathological severity. There were significant PLR differences across the outcome groups (*p* = 0.005), with notably higher values in cases of invasive carcinoma (*p* < 0.01). ROC analysis showed moderate diagnostic utility (AUC ≈ 0.72); at a PLR cutoff of ~11.9, sensitivity was 65% and specificity 81%. Conclusions: The PLR cutoff of 7.7 was associated with HPV positivity, while a higher cutoff of 11.93 was identified for predicting invasive cervical cancer. These findings support that preoperative PLR is a non-invasive, clinically relevant marker correlated with lesion severity, offering potential for preoperative risk stratification, particularly where advanced diagnostics are limited.

## 1. Introduction

Cervical cancer is the fourth most common oncological disease among women worldwide, with its highest incidence typically occurring between the ages of 30 and 35. In 2018, an estimated 570,000 new cases and 311,000 deaths were reported worldwide [1]. Despite advancements in prevention, such as widespread HPV vaccination and organized screening programs, the disease is still frequently diagnosed at advanced stages—especially in low-resource settings where access to diagnostic tools is limited [2,3,4]. Persistent infection with high-risk human papillomavirus (HPV) is the primary etiological factor, contributing to over 95% of cervical cancer cases. The disease progresses through stages (CIN1, CIN2, CIN3), and it can be identified and treated effectively if detected early [5]. Loop electrosurgical excision procedure (LEEP) is a widely utilized method for managing cervical intraepithelial neoplasia (CIN), offering both diagnostic and therapeutic benefits by excising abnormal tissue. While effective, LEEP may reveal lesions more advanced than initially predicted by cytology, underscoring the limitations of traditional cytological screening [6].

The need for accessible, cost-effective predictive tools has prompted growing interest in peripheral blood-based biomarkers, which reflect the systemic inflammatory response associated with tumor development and progression [7]. Tumor-induced immune suppression and chronic inflammation can lead to lymphopenia by impairing lymphocyte production and function. Concurrently, tumors promote leukocytosis, notably neutrophilia, through the release of pro-inflammatory cytokines such as IL-6 and granulocyte colony-stimulating factor (G-CSF), contributing to an elevated neutrophil-to-lymphocyte ratio (NLR), which is associated with poor prognosis [8]. Additionally, paraneoplastic thrombocytosis results from increased thrombopoietin production and inflammatory cytokine signaling, with a high platelet-to-lymphocyte ratio (PLR) correlating with cancer progression and metastasis [9]. The neutrophil-to-lymphocyte ratio (NLR) has garnered significant attention across various malignancies, including gynecological cancers [7,10]. More recently, the platelet-to-lymphocyte ratio—dividing the platelet count by the lymphocytes—has emerged as another promising indicator, derived from routine complete blood counts. Unlike NLR, PLR may better reflect thrombopoietic activation and immune suppression simultaneously, capturing both thrombocytic and lymphocytic responses and thereby reflecting the complex interplay between pro-tumorigenic inflammation and impaired immune surveillance [11,12]. Elevated platelet counts are often associated with tumor angiogenesis and progression, while lymphocytopenia reflects impaired anti-tumor immunity.

Despite its potential, the clinical utility of PLR in cervical pathology remains underexplored. To our knowledge, this is the first study to evaluate PLR as a preoperative risk stratification tool in patients undergoing loop electrosurgical excision procedure (LEEP) conization. In our study, we aimed to assess whether elevated preoperative PLR values correlate with histopathological outcomes following conization in patients with suspected cervical lesions. By analyzing a large cohort of cases stratified by histological grade, we aimed to investigate the association between preoperative PLR values and the presence of high-grade cervical intraepithelial neoplasia or invasive carcinoma, focusing on identifying threshold PLR levels that could distinguish benign or precancerous lesions from malignant outcomes. Furthermore, we aimed to explore the relationship between PLR, HPV status, and age, aiming to understand whether PLR might contribute to preoperative risk stratification and support clinical decision-making in cervical cancer diagnostics.

## 2. Materials and Methods

### 2.1. Patients

**Figure 1:**
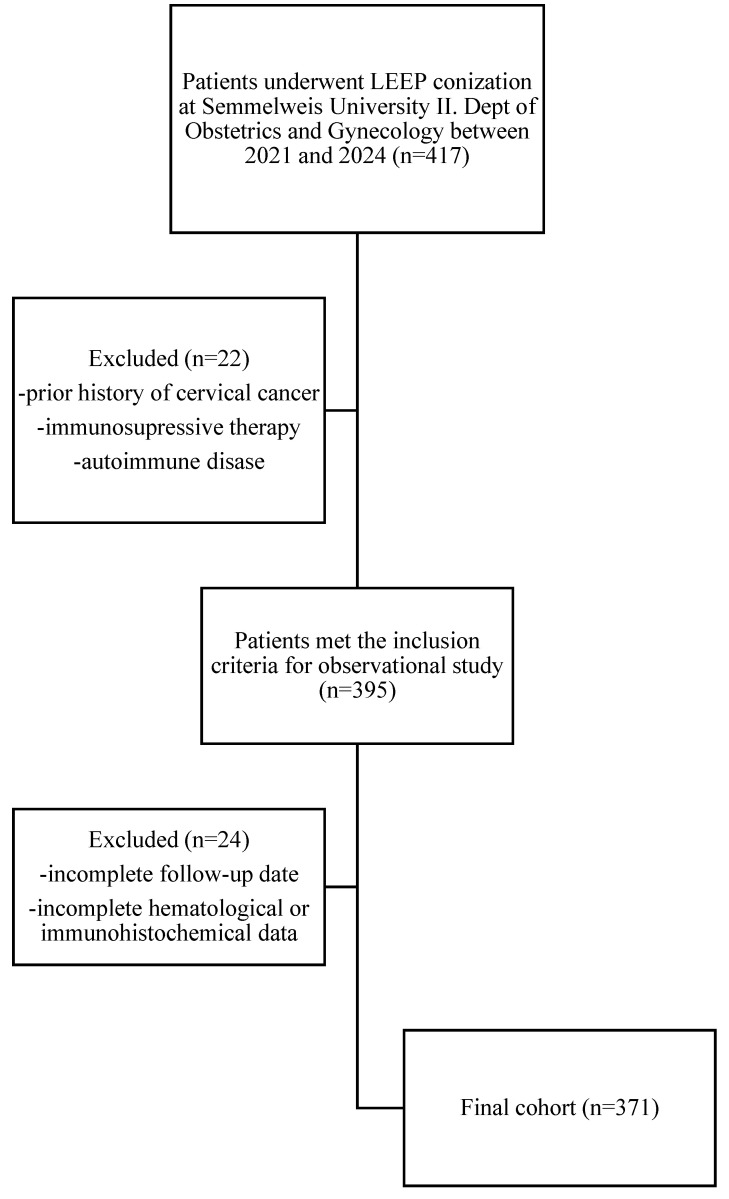
Flowchart of patient selection for the SCOPE Study.

This retrospective observational study analyzed a cohort of 371 patients as part of the SCOPE Study (Semmelweis University Conization and Inflammation Outcomes with Predictive Evaluation). The research involved an extensive review of medical records spanning from 2021 to 2024 (Figure 1). A comprehensive dataset was compiled, incorporating sociodemographic factors, gynecological history, clinical findings, and laboratory results to gain a deeper insight into patient outcomes following conization.

### 2.2. Characteristics

The sociodemographic characteristics collected in this study included patient age, and body mass index (BMI). Age was calculated by subtracting the year of birth from the year of conization. BMI was determined by using the standard formula: weight in kilograms divided by height in meters squared (kg/m^2^), based on measurements recorded during the preoperative clinical visit. Smoking status was assessed through patient self-reporting during clinical intake, and categorized as current smoker or non-smoker. Preoperative laboratory parameters focused on systemic inflammation-related biomarkers, particularly platelet and lymphocyte count, from which the platelet-to-lymphocyte ratio was calculated. All blood samples were obtained within one month before surgery to ensure temporal relevance to the conization procedure.

Screening data for cervical dysplasia included human papillomavirus (HPV) status, defined with polymerase chain reaction (PCR). Patients were categorized into four diagnostic grades based on the histopathological evaluation of their surgical conization results. Grade 1 indicated negative findings. Grade 2 encompassed low-grade lesions, including low-grade squamous intraepithelial lesion (LSIL) and atypical squamous cells of undetermined significance (ASC-US). Grade 3 included high-grade lesions, such as high-grade squamous intraepithelial lesion (HSIL), atypical squamous cells—cannot exclude HSIL (ASC-H), atypical glandular cells (AGC), and adenocarcinoma in situ (AIS). Grade 4 represented confirmed cervical carcinoma.

The study was approved by the Institutional Review Board of Semmelweis University (SE RKEB: 195/2024).

### 2.3. Data Management

Data for this study were collected as part of the SCOPE Study through a retrospective review of patient records from 2021 to 2024. All relevant patient information was systematically compiled into a dedicated research database. The dataset included comprehensive sociodemographic details (e.g., age, body mass index, smoking status), clinical history, and preoperative laboratory parameters. Complete blood count results, performed using an automated analyzer, were obtained from a single preoperative test conducted within one month prior to conization. The platelet-to-lymphocyte ratio (PLR) for each patient was calculated using the platelet counts and lymphocyte percentages derived from the complete blood count. All values are expressed in units of G/L or as percentages. High-risk human papillomavirus (HPV) DNA status results and the histopathological grade of the excised cervical lesion (from the LEEP specimen) were also documented for inclusion in the analysis. Rigorous data quality control measures were applied before statistical analysis. The compiled data were examined for completeness and consistency, with additional checks for outliers. Implausible or extreme values in continuous variables (such as PLR) were flagged using visual outlier detection (boxplot inspection) and cross-checked against the original medical records. Any detected discrepancies or missing critical data were resolved when possible, or otherwise those cases were excluded to maintain data integrity. Through this validation process, the dataset was refined to ensure reliability, resulting in a final sample of 371 patients available for analysis.

### 2.4. Statistical Analysis

All analyses were performed using IBM SPSS Statistics version 25.0 (IBM Corp., Armonk, NY, USA). Continuous variables, including PLR, exhibited non-normal distribution and were therefore summarized as median values with interquartile ranges. Categorical variables were described as frequencies and percentages. To compare PLR across the different histopathological outcome groups (categorized as Grade I through IV based on conization results), the non-parametric Kruskal–Wallis test was used. For pairwise comparisons between specific outcome categories, as well as for two-group comparisons such as PLR in high-risk HPV-positive versus HPV-negative patients, the Mann–Whitney U test was employed. A receiver operating characteristic (ROC) curve analysis was conducted to evaluate the ability of preoperative PLR to detect invasive cervical cancer. The area under the ROC curve (AUC) was calculated to quantify the discriminative performance of PLR in distinguishing patients with invasive carcinoma (histopathological Grade IV) from those with pre-invasive lesions or negative findings. An optimal PLR cutoff value for predicting invasive cancer was determined from the ROC analysis and used to define an “elevated PLR” level. A binary logistic regression model was then constructed to assess the association between an elevated PLR (above the identified cutoff) and the presence of invasive cervical cancer, with the results expressed as odds ratios (ORs) and 95% confidence intervals. All statistical tests were two-tailed, and a *p*-value < 0.05 was considered indicative of a statistically significant difference.

## 3. Results

### 3.1. Patient Characteristics

The characteristics of the study participants are summarized in Table 1. Following quality control and outlier exclusion, a total of 371 conization procedures were analyzed. The median age of the patients was 40 years, with an interquartile range of 23 to 78 years. The median body mass index (BMI) was 22.85, ranging from 14.6 to 46.4. A total of 63 patients reported being current or former smokers, while 308 were non-smokers. Regarding HPV status, 95% of patients tested HPV-positive, all of whom were infected with high-risk HPV. The median platelet-to-lymphocyte ratio (PLR) was 8.9, with an interquartile range of 3.31 to 184.21. The histopathological findings from the conization were classified into four grades: 84 (23%) were Grade I (negative), 31 (8%) were Grade II (LSIL, ASC-US), 233 (63%) were Grade III (HSIL, ASC-H, AGC, AIS), and 23 (6%) were Grade IV (cancer) (Table 2).

### 3.2. Relationship Between Smoking Habits, BMI, and PLR

To assess the influence of smoking status on PLR, a Mann–Whitney U test was performed by comparing the PLR values between smokers and non-smokers. Among the 371 patients with available data, 63 were smokers and 308 were non-smokers. The analysis revealed no statistically significant difference in PLR values between the two groups (U = 9663.0, Z = −0.557, *p* = 0.578). This suggests that PLR is not significantly affected by smoking status in this cohort and may function as a stable systemic inflammatory marker for evaluating cervical disease risk, regardless of tobacco exposure.

A correlation analysis between body mass index (BMI) and the platelet-to-lymphocyte ratio revealed a very weak but statistically significant positive relationship (Spearman’s ρ = 0.216, *p* = 0.000). Despite reaching statistical significance, the strength of this association is low, indicating that BMI exerts little to no practical influence on PLR values in this cohort. Given the low correlation coefficient, BMI is unlikely to serve as a meaningful confounding factor in the interpretation of PLR in the context of cervical lesion assessment.

### 3.3. Relationship Between Laboratory Parameters and HPV Positivity

**Figure 2:**
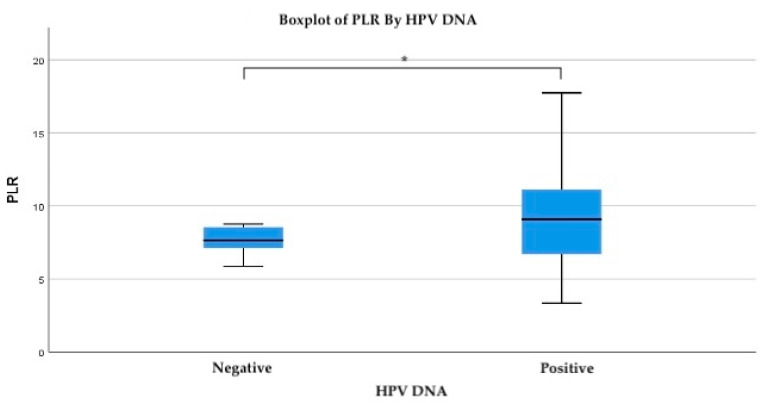
Boxplot of PLR values by HPV DNA status. Boxplot compares the distribution of PLR values between HPV DNA-negative and HPV DNA-positive patients. The median PLR was higher in HPV-positive individuals, with a wider range of values. Asterisks (*) denote a statistically significant difference between the two groups (*p* < 0.05).

To evaluate the relationship between systemic inflammatory response and HPV infection, the platelet-to-lymphocyte ratio (PLR) was analyzed in relation to HPV DNA status. The mean PLR was notably higher in HPV-positive patients (mean = 10.47) compared to HPV-negative individuals (mean = 8.05). While the standard deviation was also greater among HPV-positive cases (SD = 11.89), this reflects the broader range of inflammatory response within this group, including several extreme outliers (Figure 2).

Importantly, PLR values above 7.7 were associated with HPV positivity at the 0.05 significance level, suggesting a potential threshold above which systemic inflammation may be linked to active or persistent HPV infection. Specifically, at a PLR value of 7.761, the probability of HPV positivity was 0.29, while at a slightly lower threshold of 7.46, the association approached statistical significance (*p* = 0.052).

### 3.4. Relationship Between Laboratory Parameters and Histology Results After Conization

The relationship between the conization outcomes and the platelet-to-lymphocyte ratio (PLR) was analyzed using the Kruskal–Wallis test, which yielded a statistically significant result (*p* = 0.005). Pairwise comparisons using the Mann–Whitney U test confirmed that PLR values were significantly higher in Grade IV (cancerous) lesions compared to Grade I (*p* < 0.003), Grade II (*p* < 0.003), and Grade III (*p* = 0.001) (Table 3). However, no significant differences were observed between Grades I, II, and III, suggesting that elevated PLR is primarily associated with malignant conization outcomes. To assess the robustness of this finding, a sensitivity analysis was performed by excluding patients with extreme PLR values (>30). After exclusion, the Kruskal–Wallis test remained statistically significant (H = 13.70, *p* = 0.0033), confirming that PLR values in Grade IV lesions were significantly higher than in lower-grade groups. This supports that the association was not driven by outliers.

**Figure 3:**
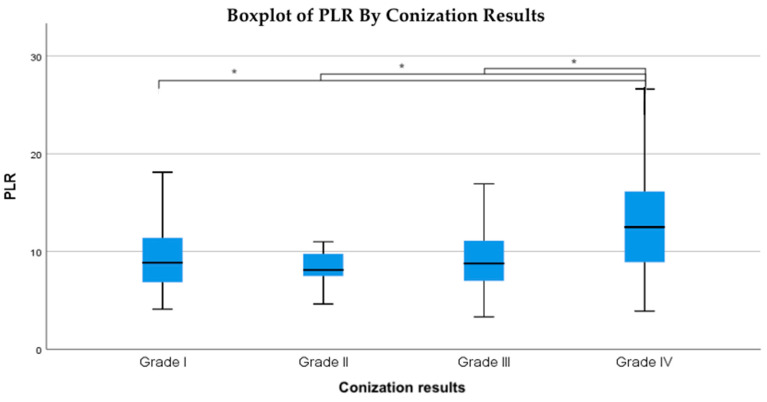
Boxplot of PLR values across histological grades. This figure illustrates the distribution of PLR values across four histopathological conization grades: Grade I (negative), Grade II (low-grade lesions), Grade III (high-grade lesions), and Grade IV (invasive carcinoma). The plot reveals a progressive increase in PLR with advancing disease severity, with Grade IV showing significantly higher PLR values compared to Grades I, II, and III. Asterisks (*) denote statistically significant differences between the groups.

The data indicate that as conization outcomes worsen, the average PLR values increase proportionally (Figure 3).

**Figure 4:**
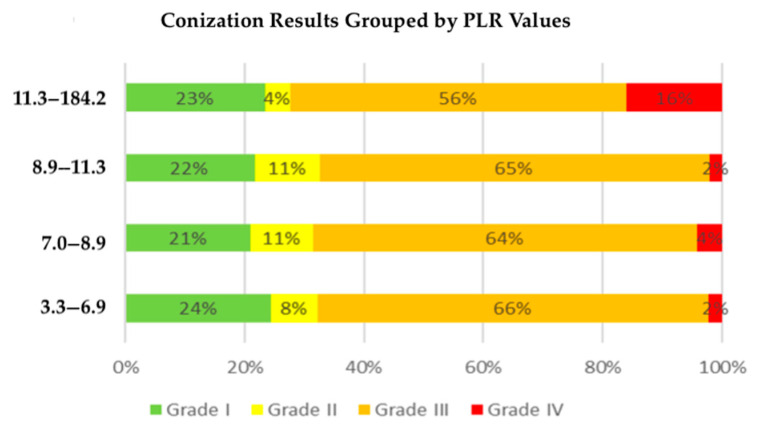
Distribution of histological grades within quartile-based PLR groups. This stacked bar chart shows the percentage of conization outcomes (Grades I–IV) within four quartile ranges (Q1: 3.3–6.9; Q2: 7.0–8.9; Q3: 8.9–11.3; Q4: 11.3–184.2) of the platelet-to-lymphocyte ratio (PLR). As PLR values increase, the proportion of patients with invasive carcinoma (Grade IV, red) also increases, whereas the proportion of benign (Grade I, green) or low-grade lesions (Grade II, light green) decreases.

Specifically, patients with Grade II (LSIL, ASC-US) conization results had a median PLR of 8.1, those with Grade III (HSIL, ASC-H, AGC, AIS) results had an average PLR of 8.7, and patients diagnosed with Grade IV cancer had an average PLR of 12.5 (Figure 4). This suggests that elevated PLR values may be indicative of worse conization results, reinforcing the potential role of systemic inflammation in cervical dysplasia progression.

### 3.5. Diagnostic Performance

**Figure 5:**
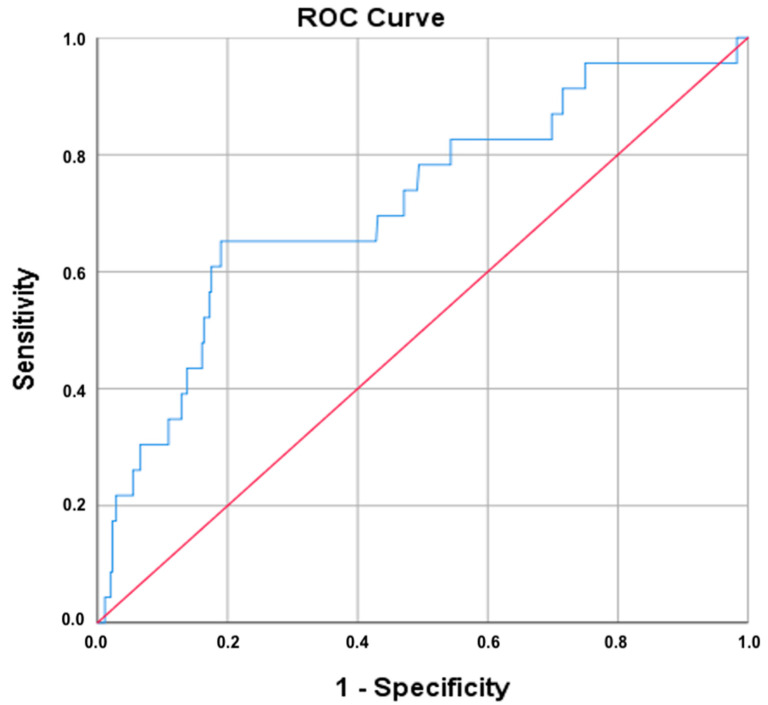
Receiver operating characteristic (ROC) curve for PLR in predicting cervical cancer. ROC curve compares the diagnostic performance of the platelet-to-lymphocyte ratio for identifying cervical cancer. Sensitivity (true-positive rate) is plotted on the *y*-axis, while 1-specificity (false-positive rate) is on the *x*-axis.

Receiver operating characteristic (ROC) analysis was conducted to evaluate the diagnostic performance of the platelet-to-lymphocyte ratio (PLR) in identifying cervical cancer. As shown in Figure 5, the area under the curve (AUC) for PLR was 0.715, suggesting a moderate-to-good discriminatory capacity. The 95% confidence interval ranged from 0.597 to 0.832, with a standard error of 0.060, and the result was statistically significant (*p* = 0.001).

Using the Youden index, the optimal PLR cut-off value was identified as ≥11.93, which yielded a sensitivity of 65.2% and a false-positive rate of 19.2% (i.e., specificity of 80.8%). The same cut-off was also selected by the Closest Top Left method, reinforcing its robustness. These results indicate that PLR may serve as a moderately effective marker for distinguishing malignant cervical lesions from less severe pathology.

Based on the optimal PLR cutoff value of 11.93, the positive predictive value (PPV) for invasive cervical carcinoma was 17.6%, while the negative predictive value (NPV) was 97.4%. These findings suggest that although elevated PLR alone has limited ability to confirm malignancy, it may be useful as a rule-out tool in clinical settings.

## 4. Discussion

Despite global health initiatives, including the World Health Organization’s call for cervical cancer elimination, cervical cancer remains a significant challenge, especially in underserved regions [13,14]. Inadequate access to early diagnostic tools means many high-risk individuals experience a delayed detection of malignancy [9,15]. Our findings address this gap by evaluating the platelet-to-lymphocyte ratio (PLR) as an available biomarker for predicting cervical malignancy in patients undergoing conization. In this study, PLR values were significantly elevated in patients whose conization surgery revealed invasive cervical cancer, compared to those with only pre-invasive lesions. Median PLR levels rose progressively with higher histopathological grades, and patients with Grade IV (invasive carcinoma) had markedly higher PLR than those with lower-grade dysplasia. This gradient supports a biological link between systemic inflammation and the progression from cervical intraepithelial neoplasia (CIN) to invasive cancer.

The elevation of PLR in HPV-positive individuals likely reflects virus-induced systemic inflammation, including increased interleukin-6 production, which stimulates thrombopoiesis and leads to reactive thrombocytosis. In contrast, in invasive cervical cancer, PLR elevation may be further amplified by tumor-associated immune suppression, characterized by lymphocyte depletion due to chronic immune exhaustion. Thus, while both conditions contribute to increased PLR, their biological underpinnings differ: inflammatory thrombocytosis predominates in HPV-related dysplasia, whereas immune dysregulation and lymphocytopenia are more pronounced in malignancy [16].

PLR values above 7.7 were associated with HPV positivity at the 0.05 significance level, suggesting a potential threshold above which systemic inflammation may be linked to active or persistent HPV infection. Notably, our ROC analysis indicated that PLR has a moderate ability to discriminate malignant from non-malignant lesions (AUC: 0.72), with an optimal PLR threshold yielding high specificity (81%) for predicting cancer. While the sensitivity at this cut-off was more modest (65%), these results suggest that an elevated PLR can serve as a warning sign of underlying malignancy in a patient with an abnormal cervical screening result.

In a recent study conducted at our institute highlighting the role of the neutrophil-to-lymphocyte ratio (NLR) in cervical cancer progression, we also found that HPV-positive individuals showed a higher mean NLR compared to HPV-negative individuals, which aligns with our results regarding PLR [10]. These findings support the importance of preoperative inflammatory ratios. Recent studies by Huang et al. have reported that patients with elevated preoperative PLR values are more likely to have significant cervical intraepithelial neoplasia (CIN) or residual disease following conization, highlighting PLR’s potential as a pre-surgical risk stratification tool for predicting recurrence or residual high-grade squamous intraepithelial lesions (HSILs) after LEEP [17,18]. Likewise, in a comparative analysis of peripheral blood counts, Wang and Dong found that cervical cancer patients had a significantly higher PLR than those with high-grade CIN or healthy controls. PLR levels increased with advancing FIGO stage and deeper stromal invasion, reflecting a clear association between elevated PLR and the presence and extent of malignancy [19]. A retrospective study on 324 women undergoing colposcopy for cervical lesions compared NLR, PLR, MLR (monocyte-to-lymphocyte ratio), and SII (systemic immune-inflammation index) across chronic cervicitis, LSIL, HSIL, and cervical cancer patients. Although each marker showed relatively low AUC (PLR: 0.582), combining them increased diagnostic sensitivity (≈69%) [20]. These concordant findings from different populations reinforce that PLR is intimately linked to cervical disease severity. 

Importantly, an elevated PLR may also capture aspects of the host’s interaction with HPV infection. Bilir et al. demonstrated that persistent high-risk HPV infection occurred more frequently in patients with a high PLR, suggesting that a pronounced inflammatory state (high platelet count) coupled with relative lymphopenia might reflect an impaired immune clearance of HPV [21]. An elevated PLR may reflect both tumor-driven inflammation and impaired immune clearance of HPV, together promoting viral persistence and lesion progression. Tumor cells and HPV-infected dysplastic cells release cytokines such as IL-6 and granulocyte colony-stimulating factor, driving up platelet production while also inducing an immunosuppressive milieu that reduces lymphocyte counts. This mechanism aligns with findings that a high PLR is associated with greater tumor burden and poorer outcomes, including reduced progression-free and overall survival in cervical cancer.

For instance, studies of patients treated for cervical carcinoma have found that those with an elevated pretreatment PLR experience worse progression-free and overall survival. An elevated PLR, especially in conjunction with a high neutrophil–lymphocyte ratio, signifies an intensified systemic inflammatory response and a hypercoagulable state that can promote cancer growth and metastasis [22].

PLR has also shown prognostic relevance in other HPV-associated malignancies. For example, in oropharyngeal squamous cell carcinoma, high PLR correlates with poorer disease-free and overall survival, reflecting similar inflammatory mechanisms to those observed in cervical cancer [23,24]. Moreover, HPV-positive oropharyngeal tumors, which generally carry a better prognosis, tend to present with lower systemic inflammation and consequently lower PLR values [25]. Similarly, in anal squamous cell carcinoma, elevated PLR before treatment has been linked to lower rates of complete response to chemoradiotherapy and worse survival. Stojanović-Ruńdić et al. found that PLR outperformed NLR in predicting treatment failure, identifying a threshold that reliably distinguished responders from non-responders [26]. These findings from multiple HPV-driven cancer types highlight PLR as a broadly applicable inflammatory biomarker, reinforcing its biological and clinical relevance.

Our study and the literature suggest that PLR is a meaningful, non-invasive indicator of malignancy risk in cervical lesions. An elevated PLR, as part of a routine blood count, could alert clinicians to patients who are more likely to harbor invasive disease despite relatively moderate cytologic or colposcopic findings. This biomarker reflects the biology of cervical cancer progression and thus has the potential to improve early detection when used alongside traditional screening and diagnostic methods.

### 4.1. Strengths and Limitations

This study’s main strength lies in its large sample size and comprehensive statistical approach, providing robust evidence for the association between elevated PLR and malignant conization outcomes. The use of multiple analytic methods and histologically confirmed diagnoses enhances the reliability and clinical relevance of our findings. PLR is an inexpensive, widely available test, making its potential application highly practical. However, limitations include the retrospective, single-center design and possible selection bias. Given the single-center design of this study, replication in a larger, multicenter cohort would substantially enhance the reliability of PLR as a biomarker.

The relatively small number of cancer cases may limit the precision of PLR cut-off values, and the moderate AUC suggests that while PLR demonstrates potential as a diagnostic marker, it should be interpreted in conjunction with other clinical indicators to improve decision-making. Additionally, we did not evaluate long-term prognostic value or integrate other biomarkers, warranting future prospective studies for validation.

The small number of invasive cancer cases represents a key limitation of this study. Although the PLR cutoff showed promising discriminatory performance, the limited sample size may have influenced the optimal threshold. Therefore, validation in larger, independent cohorts is required to confirm its reliability and clinical applicability.

As over 95% of patients in our cohort were HPV-positive, the ability to evaluate the PLR’s diagnostic performance in HPV-negative malignancies was limited. This constrains the generalizability of our findings and highlights the need for future studies that include more HPV-negative cases to assess the broader clinical utility of the PLR.

Although patients with conditions known to affect the PLR were excluded, potential confounders such as comorbidities and concomitant medications were not addressed.

### 4.2. Implications for Practice

PLR is a low-cost, widely available biomarker that could support risk stratification in cervical cancer diagnostics, especially in low-resource settings. Derived from routine blood tests, PLR may help prioritize patients with high-grade cytology or HPV positivity for more urgent colposcopic evaluation or treatment. In clinical practice, PLR could be integrated as an adjunct triage tool. For instance, HPV-positive patients with borderline or low-grade cytological findings and a PLR > 11.93 could be prioritized for immediate colposcopic evaluation, potentially improving the early detection of invasive disease while reducing unnecessary procedures in lower-risk individuals. While not a standalone diagnostic tool due to its moderate accuracy, PLR can complement existing methods and be integrated into multimodal risk models alongside markers like NLR, p16/Ki-67, and HPV genotyping; especially in resource-limited settings, clinical use could enhance early detection and personalized care, particularly where advanced diagnostic resources are limited.

## 5. Conclusions

This study demonstrates that an elevated platelet-to-lymphocyte ratio (PLR) is significantly associated with malignant histopathological outcomes in patients undergoing conization for cervical lesions. Elevated PLR values were primarily observed in patients with invasive cervical cancer, suggesting that PLR reflects both tumor-related inflammation and potential immune suppression. Given its availability through routine blood tests and its moderate diagnostic accuracy, PLR emerges as a cost-effective, non-invasive adjunct biomarker that may aid in risk stratification and the early identification of cervical malignancy—particularly in settings where access to advanced diagnostics is limited.

## Figures and Tables

**Table 1:** Characteristics of the sample. Categorical parameters are presented as n. Continuous data are presented as median (interquartile range).

Characteristics (*n* = 371)	N (Range or %)
Total	371
Median age (years)	40 (23–78)
Median BMI	22.85 (14.6–46.4)
Median PLR	8.9 (3.31–184.21)
	
Smoking status	
Smoker	63 (17%)
Non-smoker	308 (83%)
	
HPV (HR HPV) status	224
HPV-positive	212 (95%)
HPV-negative	12 (5%)
	
Conization results	371
Grade I	84 (23%)
Grade II	31 (8%)
Grade III	233 (63%)
Grade IV	23 (6%)

**Table 2:** PLR values by histological grade. Continuous variables are presented as median, range, minimum, and maximum.

Histological Grade	Median PLR	N	Range	Minimum	Maximum
Grade I	8.86	84	180.11	4.1	184.21
Grade II	8.11	31	14.05	4.62	18.67
Grade III	8.77	233	155.73	3.31	159.05
Grade IV	12.5	23	22.71	3.89	26.6
Total	8.9	371	180.9	3.31	184.21

**Table 3:** Pairwise comparisons between PLR values across histological results. This table presents pairwise comparisons of platelet-to-lymphocyte ratio (PLR) values across histopathological conization outcome grades using the Mann–Whitney U test. Asterisks (*) denote statistically significant results (*p* < 0.05).

Compared Groups	Mann–Whitney U	z-Value	*p*-Value
Grade I versus Grade II	1145.0	−0.990	0.322
Grade I versus Grade III	9295.0	−0.682	0.495
Grade II versus Grade III	3398.5	−0.533	0.594
Grade I versus Grade IV	573.5	−2.977	0.003 *
Grade II versus Grade IV	188.0	−2.948	0.003 *
Grade III versus Grade IV	1517.5	−3.430	0.001 *

## Data Availability

The data supporting the findings of this study can be obtained by contacting the corresponding author upon request.

## Abbreviations

The following abbreviations are used in this manuscript:

AGC	Atypical Glandular Cells
AIS	Adenocarcinoma In Situ
ASC-H	Atypical Squamous Cells—Cannot Exclude HSIL
ASC-US	Atypical Squamous Cells of Undetermined Significance
AUC	Area Under the Curve
BMI	Body Mass Index
CIN	Cervical Intraepithelial Neoplasia
FIGO	International Federation of Gynecology and Obstetrics
HPV	Human Papillomavirus
HSIL	High-grade Squamous Intraepithelial Lesion
LEEP	Loop Electrosurgical Excision Procedure
LMR	Lymphocyte-to-Monocyte Ratio
LSIL	Low-grade Squamous Intraepithelial Lesion
NLR	Neutrophil-to-Lymphocyte Ratio
MLR	Monocyte-to-Lymphocyte Ratio
PCR	Polymerase Chain Reaction
PLR	Platelet-to-Lymphocyte Ratio
ROC	Receiver Operating Characteristic SII- Systemic Immune-Inflammation Index
